# Supplementary material for: Sex-biased expression of enteroendocrine cell-derived hormones contributes to higher fat storage in Drosophila females
Source: eLife. 2026 Oct 1;14:RP109426. doi: 10.7554/eLife.109426 (PMC13630383; doi:10.7554/eLife.109426)
Supplement: Peer review history

# Editor report: eLife Assessment

**DOI:** 10.7554/eLife.109426.3.sa0

**Authors:** Hiromu Tanimoto

**Affiliations:** Tohoku University Japan

**Keywords:** Incomplete, Useful

This **useful** study provides a systematic comparison of sex-biased enteroendocrine hormone expression in Drosophila and suggests that gut-derived peptides may contribute to female-biased triglyceride levels. The revised manuscript includes helpful textual clarifications and an integrative model, but the evidence remains **incomplete**, because the proposed role of Tk is still over-interpreted relative to authors' stated criterion for statistical significance against both parental controls. The work will be of interest to researchers studying sex differences in metabolism, but the central mechanistic claims require either stronger experimental support or more careful qualification.

# Referee report: Reviewer #1 (Public review)

**DOI:** 10.7554/eLife.109426.3.sa1

Summary of goals:

The authors' stated goal (line 226) was to compare gene expression levels for gut hormones between males and females. As female flies contain more fat than males, they also sought to identify hormones that control this sex difference. Finally, they attempted to place their findings in the broader context of what is already known about established underlying mechanisms.

Strengths:

(1) The core research question of this work is interesting. The authors provide a reasonable hypothesis (neuro/entero-peptides may be involved) and well-designed experiments to address it.

(2) Some of the data are compelling, especially positive results that clearly implicate enteropeptides in sex-biased fat contents.

Comments on revised version:

There are small but useful improvements in the revised manuscript. Textual revisions have helped clarify some points, and I particularly appreciate the model (Figure 5). It gives a broader overview of fat storage regulation, even if new insights are limited to a generic statement that this phenomenon is complex (e.g. line 261).

One crucial sticking point is again the handling of statistics. As the authors now explain, peptide knockdown effects are significant only if the experimental group differs from both parental controls (lines 191-194). By this definition (which is indeed the field standard and I also agree with), Tk knockdown had no significant effect (Figure 3B). The authors partially acknowledge this, initially calling the result a trend (line 198), but in many other places in their manuscript (e.g. lines 258-259, line 333) including in the Abstract (line 30) they (misre)present it as if it were significant. I have a huge problem with this, and it is the reason why I evaluate the strength of the evidence as Incomplete.

Overall, I do not think it is meaningful for authors to undergo a new (second) revision if they do not carry out experiments to address key points.

# Author comment: Author response

**DOI:** 10.7554/eLife.109426.3.sa2

**Authors:** Puja Biswas, Elizabeth Rideout

**Affiliations:** The University of British Columbia Vancouver Canada; University of British Columbia Vancouver Canada

The following is the authors’ response to the original reviews.

**Public Reviews:**

**Reviewer #1 (Public review):**

Summary of goals:

The authors' stated goal (line 226) was to compare gene expression levels for gut hormones between males and females. As female flies contain more fat than males, they also sought to identify hormones that control this sex difference. Finally, they attempted to place their findings in the broader context of what is already known about established underlying mechanisms.

Strengths:

(1) The core research question of this work is interesting. The authors provide a reasonable hypothesis (neuro/entero-peptides may be involved) and well-designed experiments to address it.

(2) Some of the data are compelling, especially positive results that clearly implicate enteropeptides in sex-biased fat contents (Figures 1 and 3).

We thank the Reviewer for this overall positive assessment of our work.

Weaknesses:

(1) The greatest weakness of this work is that it falls short of providing a clear mechanism for the regulation of sex-biased fat content by AstC and Tk. By and large, feminization of neurons or enteroendocrine cells with UAS-traF did not increase fat in males (Figure 2). The authors mention that ecdysone, juvenile hormone or Sex-lethal may instead play a role (lines 258-270), but this is speculative, making this study incomplete.

Figure 2 shows pan-neuronal or EE-specific expression of the female-specific Tra isoform (*UAS-traF*) did not explain sex differences in mRNA levels of EE cell-derived factors (we did not test body fat in this figure). We therefore agree that we did not pinpoint the upstream regulator of this difference, and suggest in our revised manuscript that identifying this regulator(s) will be an important future direction of our work.

“Another important task for future studies will be to elucidate how sex differences in neuropeptide expression are established. The first step in understanding these mechanisms will be to determine which factors specify the sex bias in neuropeptide mRNA levels. Because our data shows that sex determination gene *tra* does not regulate the sex bias in neuropeptide expression in either the brain or the gut, the role of other factors that influence sexual identity and sexual differentiation must be assessed. One strong candidate is the steroid hormone ecdysone, as virgin females have higher ecdysone titers than males. Ecdysone plays a role in regulating sexual differentiation and development, and contributes to male-female differences in multiple aspects of intestinal physiology (e.g., intestinal stem cell proliferation) and brain development. Another candidate is juvenile hormone, which has been shown to regulate sexual maturation in *Drosophila* and other insects. While it remains unclear whether juvenile hormone titers differ between virgin males and females, juvenile hormone regulates many aspects of gut physiology in mated females (e.g., intestinal lipid accumulation, ISC proliferation) and influences brain development. Other than hormones, it is possible that sex determination gene Sex-lethal plays a role in regulating the sex difference in mRNA levels of EE cell-derived hormones, as *tra*-independent effects of Sex-lethal have been described in the brain.”

(2) Related to the above point, the cellular mechanisms by which AstC and Tk regulate fat content in males and females are only partially characterized. For example, knockdown of TkR99D in insulin-producing neurons (Figure 4E) but not pan-neuronally (Figure 4B) increases fat in males, but Tk itself only shows a tendency (Figure 3B). In females, the situation is even less clear: again, Tk only shows a tendency (Figure 3B), and pan-neuronal, but not IPC-specific knockdown of TkR99D decreases fat.

We thank the Reviewer for raising this point. In terms of general data interpretation, unless the ‘experimental genotype’ (e.g., cell type-specific gain/loss of a gene) shows a significant difference in gene expression or body fat (e.g., lower body fat/gene expression) from both control genotypes (UAS control, GAL4 control), the cell type-specific manipulation of a gene is not considered to have a biologically meaningful effect as it does not differ in phenotype from the parental strains.

To ensure reader clarity on this issue we added the following text:

“For these data, cell type-specific Tra overexpression was considered to have a significant effect on EE cell-expressed hormones only if the experimental genotype (e.g., *tissue-GAL4>UAS-tra^F^*) significantly differed from both parental strains (e.g., tissue-GAL4>+ and +>UAS-*tra^F^*) with the same direction of effect.”

“For all fat storage data, cell type-specific RNAi was considered to have a significant effect on fat storage only if the experimental genotype (e.g., *tissue-GAL4>UAS-RNAi*) significantly differed from both parental strains (e.g., *tissue-GAL4*>+ and +>*UAS-RNAi*) with the same direction of effect.”

Thus, in Figure 3B our data shows that gut-specific loss of Tk caused a trend toward decreased body fat in females (*p*^GAL4^=0.1109 and *p*^UAS^=0.0118) with no effect in males (*p*^GAL4^<0.0001 and *p*^UAS^=0.5704).

In Figure 4B our data shows that pan-neuronal loss of TkR99D caused a significant decrease in female body fat (*p*^GAL4^<0.0001 and *p*^UAS^<0.0001) with no effect in males (*p*^GAL4^>0.9999 and *p*^UAS^>0.9999).

In Figure 4E our data shows that IPC-specific loss of TkR99D caused a significant increase in male body fat (*p*^GAL4^<0.0001 and *p*^UAS^=0.0003) with no effect in females (*p*^GAL4^=0.0321 and *p*^UAS^=0.0724).

To summarize our findings for the reader, in our revised manuscript we added text to the Results section:

“This suggests a role for gut-derived AstC and a potential role for gut-derived Tk in regulating female body fat, whereas gut-derived AstC or Tk do not play a role in regulating male body fat.”

“These findings are interesting for several reasons. For example, in males, loss of EE cell-derived *Tk* and loss of *TkR99D* across neurons had no effect on fat storage, in contrast to the greater fat storage observed with IPC-specific *TkR99D* loss. This suggests that Tk derived from outside of the gut, and likely in the head, regulates fat storage via effects on *TkR99D* in the IPC. Future experiments will be needed to test this model, and to determine how Tk affects IPC biology. Further studies will also be needed to understand why IPC but not pan-neuronal loss of TkR99D causes an effect on body fat. Possible explanations include greater knockdown in the IPC using *Dilp2-GAL4* or that Tk mediates opposing effects on body fat via effects on additional neuron groups with pan-neuronal *TkR99D* loss. In females, more work will be needed to identify the neurons upon which Tk acts to regulate body fat, and to test the relative contributions of EE cell- and brain-derived Tk in regulating body fat.”

(3) The text sometimes misrepresents or contradicts the Results shown in the figures. UAS-traF expression in neurons or enteroendocrine cells did sometimes alter fat contents (Figure 2H, S), but the authors report that sex differences were unaffected (lines 164-166). On the other hand, although knockdown of Tk in enteroendocrine cells caused no significant effect (Figure 3B), the authors report this as a trend towards reduction (lines 182-183). This biased representation raises concerns about the interpretation of the data and the authors' conclusions.

In Figure 2 we show the effects of UAS-*traF* expression in either EE cells or in neurons on mRNA levels of EE cell-derived factors (not body fat). Figure 2H shows the effect of *UAS-traF* in EE cells on Tk mRNA levels in the head, and Figure 2S shows the effect of pan-neuronal *UAS-traF* on NPF mRNA levels in the head.

We thank the Reviewer for pointing out we should comment on the significant findings in 2H and 2S even though the direction of effect does not contribute to the sex difference in mRNA levels. In our revised manuscript we added the following text to this effect:

“However, we note that Tra expression in EE cells further augments the male bias in head *Tk* mRNA levels (Figure 2H), whereas Tra expression in female neurons paradoxically decreases *NPF* mRNA levels in the head (Figure 2S).”

(4) The authors find that in males, neuropeptide expression in the head is higher (Figure 1F-J). This may also play an important role in maintaining lower levels of fat in males, but this finding is not explored in the manuscript.

We thank the Reviewer for pointing this out.

In response to an earlier comment, one of the phrases we added to the revised manuscript was to acknowledge that the increased body fat we observed due to IPC-specific loss of TkR99D in males was likely mediated by Tk in the head, as there was no significant effect of loss of EE cell-derived Tk on body fat in males.

“These findings are interesting for several reasons. For example, in males, loss of EE cell-derived *Tk* and loss of *TkR99D* across neurons had no effect on fat storage, in contrast to the greater fat storage observed with IPC-specific *TkR99D* loss. This suggests that Tk derived from outside of the gut, and likely in the head, regulates fat storage via effects on *TkR99D* in the IPC. Future experiments will be needed to test this model, and to determine how Tk affects IPC biology.”

Appraisal of goal achievement & conclusions:

The authors were successful in identifying hormones that show sex bias in their expression and also control the male vs. female difference in fat content. However, elucidation of the relevant cellular pathways is incomplete. Additionally, some of their conclusions are not supported by the data (see Weaknesses, point 3).

Impact:

It is difficult to evaluate the impact of this study. This is in great part because the authors do not attempt to systematically place their findings about AstC/Tk in the broader context of their previous studies, which investigated the same phenomenon (Wat et al., 2021, eLife and Biswas et al., 2025, Cell Reports). As the underlying mechanisms are complex and likely redundant, it is necessary to generate a visual model to explain the pathways which regulate fat content in males and females.

We agree with the Reviewer that sex differences in fat storage are complex. We were also surprised that our findings regarding EE cell-derived hormones did not contribute to sex differences in the Akh- and insulin-producing cells. This suggests the regulation of sex differences in body fat is highly complex and involves many different factors. In our revised manuscript, we added text to this effect, and a graphical abstract to synthesize our past and new findings together into a single model.

“Interestingly, these effects were not mediated by the IPC or APC, cells that we have previously shown contribute to the sex difference in fat storage. Taken together, our data provide additional insight into the highly complex mechanism(s) by which unmated female flies achieve higher fat storage than male flies (Fig. 5).”

**Reviewer #2 (Public review):**

Summary:

This manuscript by Biswas and Rideout investigates sex differences in the expression and function of hormones derived from Drosophila enteroendocrine cells (EE). The authors report that while whole-body and head expression of several EE hormones (AstA, AstC, Tk, NPF, Dh31) is male-biased, gut-specific expression of AstC, Tk, and NPF is female-biased. Intriguingly, this sex-specific effect is not dependent on Tra - a surprising and important result. The authors then used an RNAi-based approach to demonstrate that gut-derived AstC and Tk promote fat storage specifically in females. Similar effects are observed when their receptors are knocked down in neurons. In addition, the authors were able to demonstrate that while Tk promotes female body fat via the insulin-producing cells. Together, these findings suggest that EE cell-derived hormones contribute to sex-specific fat storage regulation.

We thank the Reviewer for their positive assessment of our paper.

Strengths:

Overall, I find the paper quite interesting. While the findings are brief, they reveal novel aspects of the sex-specific lipid storage program that I believe are important. As noted by the authors in the discussion, there are many open questions, including how these neuronal effects translate into systemic sex-specific regulation of lipid storage. Regardless, I find the results to be convincing - this paper will serve as the launching point of many future studies.

Weaknesses:

My main criticisms are focused on two points:

(1) If the sex specific differences are eliminated by tra overexpression, what else might be responsible? As the authors note, the differences in 20E titers might be responsible. I would encourage the authors to simply feed adult flies with food containing 20E and determine if this alters sex-specific 20E expression.

We agree that there are many candidates (e.g., ecdysone, juvenile hormone) that might contribute to sex differences in mRNA levels of EE cell-derived hormones. We suggest this is an important future direction of our work.

“Another important task for future studies will be to elucidate how sex differences in neuropeptide expression are established. The first step in understanding these mechanisms will be to determine which factors specify the sex bias in neuropeptide mRNA levels. Because our data shows that sex determination gene *tra* does not regulate the sex bias in neuropeptide expression in either the brain or the gut, the role of other factors that influence sexual identity and sexual differentiation must be assessed. One strong candidate is the steroid hormone ecdysone, as virgin females have higher ecdysone titers than males. Ecdysone plays a role in regulating sexual differentiation and development, and contributes to male-female differences in multiple aspects of intestinal physiology (e.g., intestinal stem cell proliferation) and brain development. Another candidate is juvenile hormone, which has been shown to regulate sexual maturation in *Drosophila* and other insects. While it remains unclear whether juvenile hormone titers differ between virgin males and females, juvenile hormone regulates many aspects of gut physiology in mated females (e.g., intestinal lipid accumulation, ISC proliferation) and influences brain development. Other than hormones, it is possible that sex determination gene Sex-lethal plays a role in regulating the sex difference in mRNA levels of EE cell-derived hormones, as *tra*-independent effects of Sex-lethal have been described in the brain.”

(2) I'm quite intrigued by the discovery that Tra does not eliminate the sex-specific differences. There are quite a few recent studies demonstrating that fruitless influences sex-specific neuronal function - here to I would encourage the authors to examine whether this aspect of the sex-determination pathway is involved in the lipid accumulation phenotype.

We thank the Reviewer for raising this point. Transcripts derived from the *fruitless*-P1 promoter, which is largely responsible for the production of male-specific Fru^M^ proteins in the CNS, are spliced by Tra. Therefore, while we cannot definitively rule out a role for fruitless, it is less likely given that the Tra expression in males (which would eliminate Fru^M^ proteins in males) did not have a significant effect. In the revised manuscript, we added text to clarify this important point.

“Future studies will also need to test additional members of the sex determination pathway. While sex differences in expression of EE cell-derived hormones does not involve *tra*, and is therefore unlikely to involve known *tra* targets such as *fruitless*, without further experiments we cannot fully rule out these additional sex determination pathway members.”

**Reviewer #1 (Recommendations for the authors):**

(1) The authors should explain why they focused on AstA, AstC, Tk, NPF and Dh31 but not Bursicon, CCHamides 1 and 2, and sNPF, especially since the latter four are also important entero-peptides.

We thank the Reviewer for raising this point. In our revised manuscript we clarify that evaluating sex differences in all EE cell-derived hormones will be an important future direction of our work.

“In particular, we focused on hormones known to influence whole-body fat metabolism, though an important future direction of this work will be to assess sex differences in all EE cell-expressed hormones.”

(2) The authors initially compare peptide gene expression in males vs. females (Figure 1), but all subsequent comparisons (Figures 2-4) are experimental group vs. controls. It is necessary to directly compare males vs. females for these experiments as well, since the sex-biased difference is the focus of the paper. This may also help with variable performance of controls for some experiments (e.g. Figure 2), which makes interpreting these data difficult.

We thank the Reviewer for making this point. In terms of general data interpretation, as with our response to an earlier point, unless the ‘experimental genotype’ (e.g., cell type-specific gain/loss of a gene) shows a significant difference in gene expression or body fat (e.g., lower body fat) from both control genotypes (UAS control, GAL4 control), the cell type-specific manipulation of a gene is not considered to have a biologically meaningful effect as it does not differ in phenotype from the parental strains.

To ensure reader clarity on this issue we added the following text to the Results section:

“For all fat storage data, cell type-specific RNAi was considered to have a significant effect on fat storage only if the experimental genotype (e.g., *tissue-GAL4>UAS-RNAi*) significantly differed from both parental strains (e.g., *tissue-GAL4>+* and *+>UAS-RNAi*) with the same direction of effect.”

In terms of comparing the sexes, all of our analyses used a two-way ANOVA and tested for a sex:genotype interaction. This allowed us to test whether males and females showed a statistically distinct response to the different genetic manipulations. To ensure clarity for readers, we include p-values for all the sex:genotype interactions in figure legends.

(3) The organization of Figure 1 is unintuitive because the authors change the order of peptides in the last row of panels (Figure 1 K-O). The authors should keep the same order, so that every column corresponds to the same peptide, to make the figure easier for readers to follow.

We thank the Reviewer for pointing out that we should make every row the same order of EE cell-derived peptides. We made this change in our revised manuscript.

(4) The authors should explain why mRNA levels in whole-body samples are so highly skewed towards males (sometimes approaching 3-fold expression), whereas in the constituting tissues (head, guts), the differences are much milder and also in opposite directions. How do the big differences in favor of males in Figure 1A-E come about? Does the inclusion of the VNC skew expression levels so much?

We thank the Reviewer for suggesting we clarify several points around the anatomical focus of sex differences in mRNA levels of EE cell-derived hormones. In our revised manuscript we explain that while male-biased mRNA levels in heads suggest that sex-biased expression in whole bodies may be attributed to expression in heads, that other tissues may contribute to the male-biased expression. We further state this is an interesting area for future investigation.

“For most peptides, the male bias was due to a higher mRNA level in the head and not the fat body (Figure S1A-E); however, *TkR99D* mRNA levels were higher in male fat bodies with no difference in head mRNA levels (Figure S1C). We therefore cannot rule out a contribution of additional anatomical sites to the male bias in expression of EE cell-expressed hormones, which is an interesting area for future investigation.”

(5) The authors use voila-GAL4 as a driver for enteroendocrine cells, but this line is also expressed in sensory cells. The authors should at least mention the expression pattern of this line at first mention (line 165).

We thank the Reviewer for raising this point, we added text to this effect in the revised manuscript:

“We found that sex differences in mRNA levels of *AstA*, *AstC*, *Tk*, *NPF*, and *Dh31* were unaffected when we used either *voila-GAL4* (Figure 2A-2J) which expresses in EE and sensory cells, or *elav-GAL4* (Figure 2K-2T) which expresses in neurons and neuropeptide-producing cells, to drive Tra expression in these cells.”

(6) Figure legends for Figures 2, 3 and 4 should be simplified and condensed to more concisely describe the panels. There is a lot of redundant repetition, which can easily be avoided by organizing the panels into groups (for example, in Figure 2, A-E should get a single legend entry rather than separate ones).

We thank the Reviewer for this suggestion, we shortened our legends in the revised manuscript.

(7) The authors refer to triglyceride contents as 'fat storage', but triglycerides can also be carried through the hemolymph via lipoproteins. The authors should use a more factual expression like 'total triglycerides'.

We thank the Reviewer for this comment. Circulating lipoproteins in *Drosophila* carry primarily diacylglycerol, phosphatidylethanolamine, and sterol, with only a small fraction of triacylglycerol (PMID 22844248). Nevertheless, to ensure we are clear we added text in the Methods section to clarify that “fat storage” refers to whole-body triacylglycerol.

“Triglyceride is the main form of stored fat in the body, with very little in the circulation. We therefore refer to whole-body triglyceride levels as ‘fat storage’ or ‘body fat’.”

(8) The authors should justify their use of unmated flies for their experiments (line 324) and comment if they expect similar findings and mechanisms in mated flies, especially since nutritional and energy demands are greater in mated females.

We added text to the methods to justify our use of unmated females to uncover the genetic mechanisms that contribute to sex differences in body fat.

“We used unmated flies to identify genetic factors that regulate the sex difference in body fat; mated females were not used to avoid mating-induced changes in physiology mediated by additional factors (e.g., Sex-peptide) and behavioral changes due to altered food preferences.”

(9) Are there any additional AstC and/or Tk receptors that could also play a role? The authors should comment on why they focused on AstC-R2 and TkR99D alone.

We thank the Reviewer for this interesting point. We added text in our revised manuscript to acknowledge that we tested the primary known receptors for AstC and Tk, other receptors may contribute to their effects.

“We therefore predicted that loss of *AstC-R2* and *TkR99D* in these cells would reproduce the reduced fat storage we observed in females with loss of EE cell-derived AstC and Tk, though we cannot fully rule out effects of Tk and AstC mediated by other receptors as we did not test these additional receptors.”

(10) The authors cite Song et al. 2014 to justify using R57C10-GAL80 to restrict expression patterns to the gut (lines 177-179), but upon checking that paper,r I could not find that Song et al. used this approach. Please scrutinize this and remove the reference if it is incorrect.

We thank the Reviewer for pointing out that Song et al. did not specify how they achieved gut-specific Tk-GAL4; we removed this reference.

**Reviewer #2 (Recommendations for the authors):**

(1) Line 70 - the statement "In males, body fat is maintained..." seems too generic. I would suggest a small edit - "In males, body fat levels are maintained...".

This is a good suggestion, thank you, we made the appropriate adjustment.

“In males, body fat levels are maintained by higher expression and activity of two catabolic pathways that promote fat breakdown.”

(2) Lines 78-81 - These statements suggest an either/or scenario, but I assume this is more a function of balance and equilibrium, where females have more ISS signaling that maintains elevated fat, while bmm pushes homeostasis in males toward catabolism. The authors should include more nuanced statements.

We thank the Reviewer for this suggestion. In our revised manuscript we adjusted the text as follows:

“Together, these studies have defined a model of the sex difference in fat storage in which females maintain higher levels of fat storage in part due to a higher relative activity level for anabolic pathway IIS, whereas males have lower fat storage due to higher relative activity of catabolic effectors such as *bmm* and Akh.”

(3) Please provide all RRID numbers for the listed BDSC strains - the RRID numbers can be found at the bottom of the BDSC page for each strain.

We thank the Reviewer for this suggestion, we added the RRID to the Methods.

(4) Please cite the most recent FlyBase manuscript published in Genetics. Ideally, a statement under the fly husbandry section noting that Flybase was used as a resource throughout the study.

Thank you for this suggestion, we made the requested change to properly acknowledge this critical community resource.

“We acknowledge FlyBase as an essential resource providing genetic, genomic, and functional data and tools that supported this study.”
